# Immune response of heterologous recombinant antigenic protein of viral hemorrhagic septicemia virus (VHSV) in mice

**DOI:** 10.1080/19768354.2019.1575904

**Published:** 2019-02-08

**Authors:** Chunha Shin, Yangjoo Kang, Heui-Soo Kim, Yong Kyoo Shin, Kisung Ko

**Affiliations:** a Department of Medicine, College of Medicine, Chung-Ang University, Seoul, South Korea; b Department of Biological Sciences, College of Natural Sciences, Pusan National University, Busan, South Korea; c Department of Pharmacology, College of Medicine, Chung-Ang University, Seoul, South Korea

**Keywords:** Plant expression system, vaccine, VHSV, VHSVG-FcK

## Abstract

Viral hemorrhagic septicemia (VHS) is an important infectious disease in fish worldwide caused by viral hemorrhagic septicemia virus (VHSV). VHSV is the causative agent of serious systemic diseases in fish, affecting a number of teleost fish species. In this study, VHSV glycoprotein (G), including its epitope, as a subunit vaccine candidate, was expressed in tobacco plant (*Nicotiana tabacum*). The recombinant gene, VHSVG, was fused to the immunoglobulin Fc fragment and extended with the endoplasmic reticulum (ER) retention signal (KDEL) to generate VHSVG-FcK. The recombinant expression vector for VHSVG-FcK was transferred into *Agrobacterium tumefaciens* (LBA4404), and plant transformation was conducted *N. tabacum*. Polymerase chain reaction (PCR) was performed to confirm gene insertion and VHSVG-FcK protein expression was confirmed by immunoblot analysis. VHSVG-FcK protein was successfully purified from tobacco plant leaves. Furthermore, ELISA analysis showed that mice serum immunized with the plant-derived VHSVG-FcK (VHSVGP-FcK) had a high absorbance against VHSVG-FcK, indicating that the plant-derived recombinant subunit vaccine protein VHSVG-FcK can induce immune response. Taken together, this recombinant vaccine protein can be expressed in plant expression systems and can be appropriately assembled to be functional in immunogenicity.

## Introduction

Viral hemorrhagic septicemia (VHS) is serious infectious disease in fish worldwide caused by viral hemorrhagic septicemia virus (VHSV) (Walker and Winton 2010). VHSV is the causative agent of serious systemic diseases in fish, affecting a number of teleost fish species, including the olive flounder (*Paralichthys olivaceus*) (Skall et al. 2005). VHSV belongs to the *Novirhabdovirus* genus of the *Rhabdoviridae* family and has a negative-sense, single-stranded RNA genome (Caipang et al. 2003). The virus genome is composed of six parts: nucleoprotein (N), phosphoprotein (P), matrix protein (M), glycoprotein (G), RNA polymerase (L), and non-structural viral protein (NV) (Schu et al. 1999). Among the components, the glycoprotein (G) is located on the surface of the virus and can induce immune response because of the presence of epitopes (Coll 1995; Lorenzen et al. 1999; Byon et al. 2006).

To date, fish are treated with excessive antibiotics in aquaculture to manage the fish disease problem; this can lead to various side effects and increase the cost (Boxall 2004; Cabello 2006). Application of antibiotics to fish culture systems is not an environmentally friendly approach and the antibiotics can be transferred to humans via fish (Kemper 2008). Therefore, it is necessary to develop vaccines that can effectively prevent viral diseases in fish. Recently, several studies are being conducted on the development of fish vaccines (Gudding and Van Muiswinkel 2013). Several vaccines are available, such as an attenuated viral vaccine, bacterial vaccine, DNA vaccine, recombinant subunit vaccine, and virus-like particle vaccine (Lorenzen et al. 1993; Biering et al. 2005; Håstein et al. 2005; Lorenzen and LaPatra 2005; Crisci et al. 2012). Among these, the recombinant subunit vaccine is produced by using the virus glycoprotein-that contains the causative immunogenic part of the disease in a heterologous expression system. In addition, this vaccine can efficiently induce immune responses (Lecocq-Xhonneux et al. 1994; Brun et al. 2011).

In particular, to produce the recombinant protein, the Escherichia coli system or mammalian-derived cell system has been used. However, existing approaches have some limitations such as high cost, function, and safety issues (Zhu 2012; Rosano and Ceccarelli 2014). In the E. coli bacterial expression system, the prokaryotic cell cannot perform the post-translational modifications, such a glycosylation, and thus recombinant glycoproteins produced from *E. coli* cannot function effectively (Yin et al. 2007). The mammalian cell expression system is expensive and can potentially be contaminated by human pathogens (Twyman et al. 2003). However, plants can be easily cultivated for scale-up production, resulting in lower production costs without any human pathogenic contaminant concerns (Sharma and Sharma 2009; Obembe et al. 2011). Plants have post-translational modification and glycosylation processes similar to humans (Gomord and Faye 2004; Walsh and Jefferis 2006; Arcalis et al. 2013). Because of the many advantages, we used the tobacco plant expression system for the expression and purification of the recombinant vaccine candidate protein of VHSV.

In this study, the plant expression system was established for the production of the VHSV vaccine candidate to determine in in vivo animal. The VHSV glycoprotein (G) fused to the immunoglobulin Fc fragment (VHSVG-Fc) fusion protein was expressed in the tobacco plant. Its gene insertion, expression, purification, and immunofunction of VHSVG-Fc protein were investigated in tobacco plant.

## Materials and methods

### Plasmid construction

**Figure 1.**
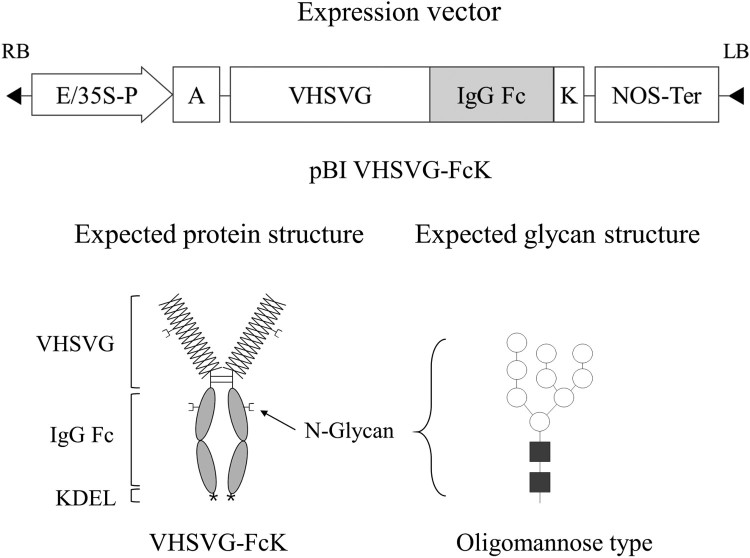
Schematic diagram of plant expression vector for VHSVG-FcK protein. VHSVG-FcK gene expression cassette was introduced to plant expression vector pBI121. E/35S-P, the Cauliflower mosaic virus 35S promoter with duplicated enhancer region; A, an alfalfa mosaic virus untranslated leader sequence (AMV) of RNA4; K, endoplasmic reticulum retention signal (KDEL); NOS-Ter, the nopaline synthase gene terminator. Expected protein structure of the recombinant fusion protein VHSVG-FcK: overlapped X shape bar, VHSVG; white oval region, Fc; and spring-shaped region, KDEL. Expected glycan structure: the symbols of the glycan structures are as follows: *N*-acetylglucosamine, black square; mannose, white circle.

The gene encoding VHSV glycoprotein (G), including epitopes, was fused to the human IgG Fc fragment extended with KDEL, the endoplasmic reticulum (ER) retention signal, to generate VHSVG-FcK (Figure 1). VHSVG-FcK was cloned under the control of the enhanced cauliflower mosaic virus (CaMV) constitutive 35S promoter with the untranslated leader sequence of alfalfa mosaic virus RNA 4 (AMV) (Figure 1). The expression cassette was cloned in the plant binary vector pBI121 by *Hind*III and *EcoR*I to produce pBI VHSVG-FcK and transferred into DH5α *E. coli* cells.

### Agrobacterium-mediated plant transformation

**Figure 2.**
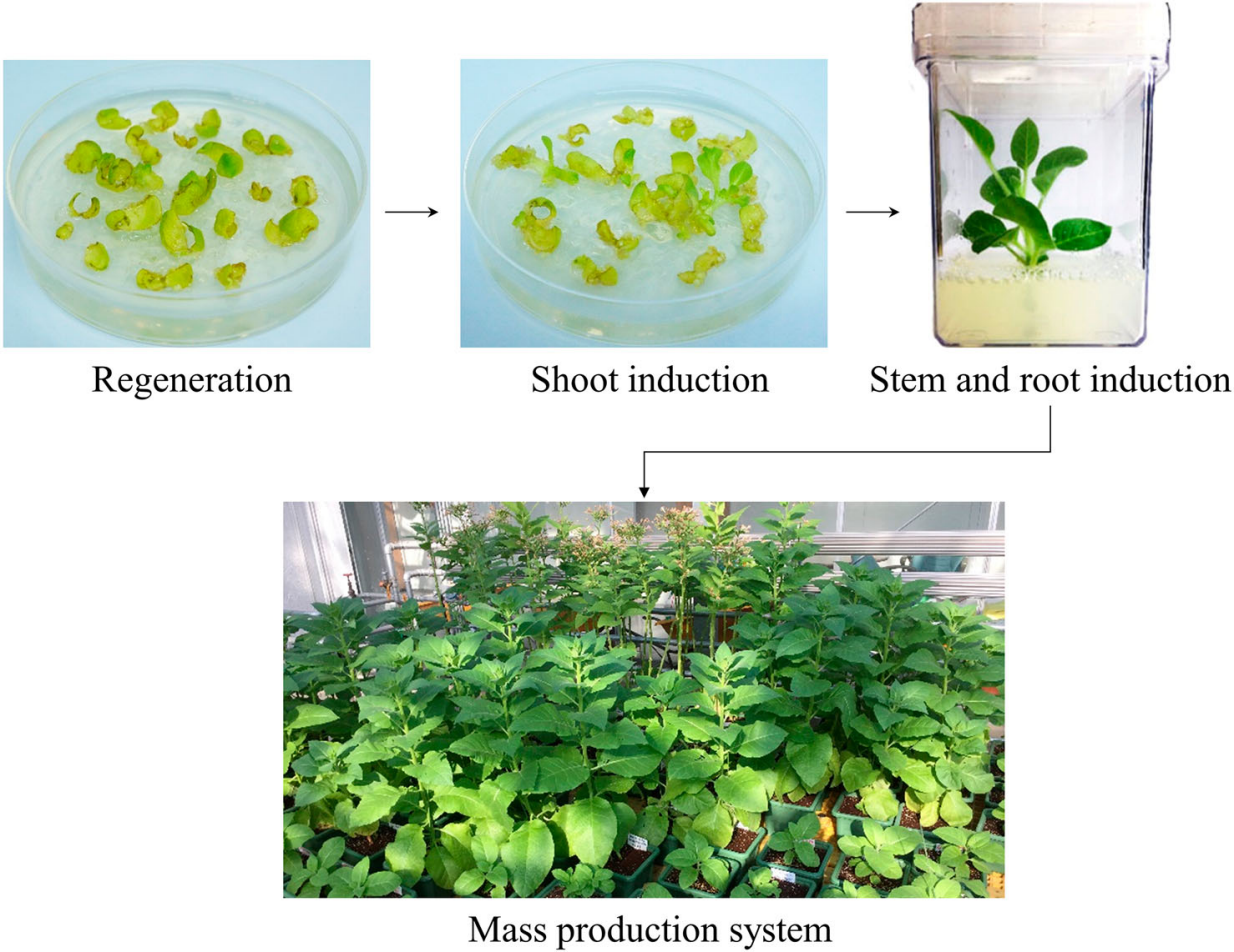
Schematic diagram of production process of VHSVG-FcK transgenic plants. After the *Agrobacterium*-mediated plant transformation, plants were regenerated under kanamycin and cefotaxime pressure and shoots were induced from callus. Shoots were transferred to magenta vessel GA-7 and stem and root were induced in kanamycin cefotaxime containing media. Transgenic plants expressing VHSVG-FcK were selected to grow for mass production.

The recombinant expression vector pBI VHSVG-FcK was transferred into *Agrobacterium tumefaciens* (LBA4404) using electroporation method. Plant transformation was conducted via the *Agrobacterium*-mediated leaf disc method using tobacco plant (*Nicotiana tabacum*) (Lu et al. 2012). The inoculated explants were placed on Murashige and Skoog (MS) medium [30 g/L of sucrose, 6 g/L of phyto agar, and 4.8 g/L of MS B5 vitamin (Duchefa Biochemie, Haarlem, Netherlands)] containing 100 mg/L of kanamycin and 250 mg/L of cefotaxime. All plants were grown in a chamber under constant temperature (23°C) and maintained in a 16-h light and 8-h dark cycle. All leaf explants were subcultured every week and developed to callus over time (Figure 2). The regenerated shoots were removed from the callus and placed for stem and root induction in magenta vessel GA-7 (Sigma, St. Louis, MO) containing MS medium with 100 mg/L of kanamycin and 250 mg/L of cefotaxime (Figure 2).

### Genomic DNA extraction and PCR

Genomic DNA was extracted from 100 mg of transgenic and non-transgenic plant leaves. The isolated genomic DNA was analyzed by polymerase chain reaction (PCR) using a DNA extraction kit (iNtRON Biotechnology, Seoul, Korea) according to the manufacturer’s recommendations. The extracted genomic DNA was amplified by PCR to confirm the recombinant VHSVG-FcK gene insertion and the following primer pairs were used: forward primer, 5′-GTC GAC ATG GAA TGG AAT ACT TTT TC-3′, and reverse primer, 5′-GCC AAA TGT TTG AAC GAT CGG-3′. PCR was performed under the following conditions: initial denaturation at 94°C for 2 min, denaturation at 94°C for 20 s, annealing at 59°C for 30 s, extension at 72°C for 2 min, and final extension at 72°C for 5 min. The pBI VHSVG-FcK vector was used as a positive control and the genomic DNA fragment from non-transgenic tobacco plant was used as a negative control. The expected size of VHSVG-FcK gene was 2286 bp.

### Immunoblot analysis

Leaves (100 mg) of transgenic plant expressing VHSVG-FcK or non-transgenic plant were collected and homogenized with 300 µL of 1X PBS (137 mM NaCl, 10 mM Na_2_HPO_4_, 2.7 mM KCl, and 2 mM KH_2_PO_4_) to extract the total soluble proteins. A volume of 16 µL of grinded samples were mixed with the sample buffer (1 M Tris-HCl, 50% glycerol, 10% SDS, 5% 2-mercaptoethanol, and 0.1% bromophenol blue) and loaded on 10% sodium dodecyl sulfate (SDS) polyacrylamide gel. Proteins were separated by SDS-polyacrylamide gel electrophoresis and transferred to a nitrocellulose membrane (Millipore Corp, Billerica, MA). The membrane was blocked with 5% skim milk (Sigma, St. Louis, MO) in TBS-T buffer [1X TBS plus 0.5% (v/v) Tween 20] for 1 h 30 min at room temperature. The blot was incubated for 2 h at room temperature with horseradish peroxidase (HRP)-conjugated goat anti-human IgG, Fcγ fragment antibody (Jackson ImmunoResearch, West Grove, PA) diluted in blocking buffer at 1:5000 and then incubated for 2 h at room temperature. The blot was then incubated for 2 h at room temperature with rabbit anti-VHSV antibody (Amsbio, Abingdon, UK) diluted in blocking buffer at 1:1000. Lastly, the blot was treated with HRP-conjugated anti-rabbit IgG (H + L) antibody (Bethyl, Montgomery, TX) diluted in blocking buffer at 1:5000 for 1 h 30 min. The protein bands were detected using the chemiluminescence substrate (Bio-Rad, Hercules, CA) and the signal was visualized by exposing the membrane to an X-ray film (Fuji, Tokyo, Japan). The non-transgenic plant sample was used as a negative control. The expected VHSVG-FcK protein size was 80 kDa.

### Purification of recombinant VHSVG-FcK protein from plant leaf

Transgenic tobacco plants expressing the VHSVG-FcK protein were transferred to pots and grown in a greenhouse (Figure 2). The leaves were harvested after 1 month. The 250 g of harvested leaves were ground in an HR2094 aluminum blender (Philips, Seoul, Korea) with extraction buffer (37.5 mM Tris-HCl pH 7.5, 50 mM NaCl, 15 mM EDTA, 75 mM sodium citrate, and 0.2% sodium thiosulfate) and centrifuged at 8800*g* for 30 min at 4°C. The supernatant was filtered with a Miracloth (Biosciences, La Jolla, CA). The filtered supernatant was adjusted to pH 5.1 with acetic acid (pH 2.4) and centrifuged at 10,200*g* for 30 min at 4°C. After centrifugation, the supernatant solution was readjusted to pH 7.0 using 3 M Tris-HCl and ammonium sulfate was added to 8% saturation. After the 2 h incubation at 4°C, the solution was centrifuged at 8800*g* at 4°C and ammonium sulfate was added to the supernatant to 22.6% saturation. After overnight incubation at 4°C, the solution was centrifuged at 8800*g* for 30 min at 4°C and the precipitate was resuspended in extraction buffer to one-tenth of the original volume. The final solution was centrifuged at 10,200*g* for 30 min at 4°C and the supernatant was filtered through a 0.45-μm filter (Millipore, Bedford, MA). The obtained sample was applied to a protein A Sepharose 4 Fast Flow (GE Healthcare, Sweden, NJ) and performed according to the manufacturer’s recommendations. SDS-PAGE was performed for the visualization of purified samples. The gel was stained with Coomassie brilliant blue staining solution (10% acetic acid, 30% methanol, and 0.05% Coomassie blue R-250) for 30 min at room temperature. The gel was destained with destaining solution (10% acetic acid and 30% methanol) through a 3 times buffer exchange for 1 h 30 min.

### Immunization of plant-derived VHSVG-FcK protein in mice

All mice experiments were approved by the Institutional Animal Care and Use Committee (IACUC) at Chung-Ang University, Seoul, Korea (Approval number: 2017-00050). Plant-derived VHSVG-FcK (VHSVG^P^-FcK) protein and 1X PBS used as a negative control were administered with an adjuvant (aluminum hydroxide) to mice through intraperitoneal injection. The samples were injected into 8-week-old female BALB/c mice three times with 10 μg of VHSVG^P^-FcK and 1X PBS with a total volume of 300 μl at intervals of two weeks. Blood sample collection was performed 10 days after the second immunization and 10 days after the third immunization by the retro-orbital sinus bleeding method.

### ELISA for confirmation of immune response of VHSVG^P^-FcK in mice

Purified VHSVG^P^-FcK sample was diluted in 0.2 M sodium carbonate/sodium bicarbonate buffer and applied to MaxiSorp 96-well plates (Nunc, Rochester, NY) at a concentration of 100 ng. The ELISA plates were incubated overnight at 4°C and washed with 1X PBS-T [1X PBS plus 0.5% (v/v) Tween 20]. The plates were blocked using 5% skim milk in 1X PBS-T (Sigma, St. Louis, MO) for 2 h at room temperature. Half serial diluted mouse serum made from 1 μl was applied to each well as a primary antibody for 2 h at room temperature. HRP-conjugated anti-mouse IgG Fc antibody (Jackson, West Grove, PA) as a secondary antibody was added to the plates. After 2 h incubation, TMB substrate solution (Seracare, Milford, MA) was added to the sample for 5 min, and the reaction was stopped by TMB stop solution (Seracare, Milford, MA). The 96-well plate was read by the ELISA reader Epoch (Biotek, Winooski, VT) at an absorbance of 450 nm.

## Results

### PCR confirmation of VHSVG-FcK gene insertion in plant

**Figure 3.**
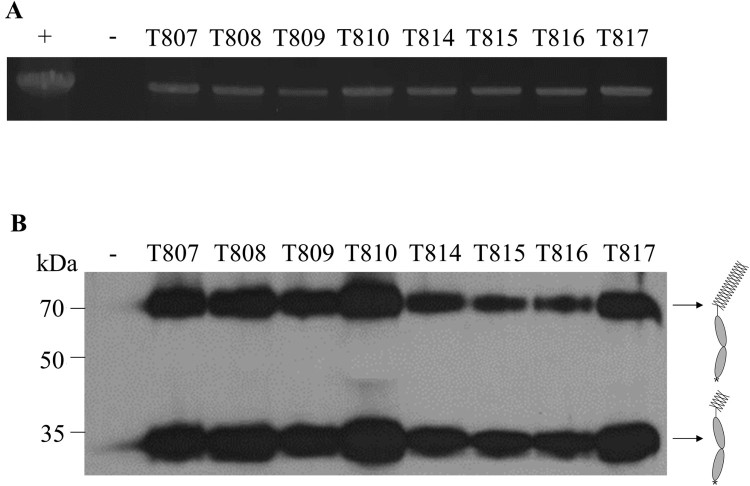
Confirmation of VHSVG-FcK gene insertion and protein expression. (A) Polymerase chain reaction (PCR) analysis was performed to confirm the VHSVG-FcK gene existence. The genomic DNA, isolated from fresh leaf, was amplified and target gene was confirmed by 1% agarose gel electrophoresis. VHSVG-FcK (2286 bp): lane 1, positive control (+), pBI VHSVG-FcK recombinant vector in DH5α competent cell; lane 2, negative control (−), genomic DNA fragment from non-transgenic tobacco plants; lane 3–10, genomic DNA fragments of transgenic plants. The 3 μl of sample was loaded on each well. (B) Immunoblot analysis was performed to confirm VHSVG-FcK protein expression in transgenic tobacco plants. Leaf samples from transgenic plants were ground with 1X PBS and 20 μl of samples were loaded on each well. VHSVG-FcK (>70 kDa) was detected with HRP-conjugated goat anti-human IgG Fc antibodies. Lane 1, negative control (−), non-transgenic tobacco plants; Lane 2–9, transgenic tobacco plants. Expected protein structures were illustrated with arrow (Right).

*Agrobacterium*-mediated transformation was conducted three times to obtain transgenic lines expressing VHSVG-FcK. Each transformation event produced three cocultivation plates containing 20 leaf cut pieces for each plate. From each transformation event, 10, 9, and 7 regenerated shoots were obtained on regeneration media. The transformants were transplanted to root-inducing media containing kanamycin antibiotic. In around two weeks after transplanting, new leaf and root growth were observed from transformants. PCR amplification of genomic DNA extracted from leaves of the putative transgenic tobacco plant was performed to confirm the VHSVG-FcK gene insertion (Figure 3(A)). The PCR products were separated on a 1% agarose gel (Figure 3(A)). The expected size of the amplified PCR band was 2286 bp. The PCR amplified band for VHSVG-FcK was detected in all transformants with healthy leaf and root growth (Figure 2). T811, T812, and T813 were transferred to root-inducing media but did not survive without any root induction. No PCR band was detected in the genomic DNA sample of the non-transgenic plant (NT) (Figure 3(A)).

### Immunoblot analysis for the confirmation of VHSVG-FcK protein expression

In order to confirm VHSVG-FcK protein expression in transgenic plants, immunoblot analysis was conducted with anti-human IgG Fc antibody conjugated horseradish peroxidase as a secondary antibody (Figure 3(B)). The expected protein band size was 80 kDa. The putative VHSVG-FcK protein band was detected at approximately >70 kDa in all tested transgenic plant samples. However, the band density of each transgenic plant varied. Among the tested transgenic lines, T810 showed the strongest density, whereas T815 and T816 had the weakest density. Thus, T810 was selected for mass production. No protein band was detected in the non-transgenic plant as a negative control. Non-specific protein bands (35 kDa) were detected in all tested transgenic plants. These had similar or slightly more prominent protein band densities compared to the >70 to 80 kDa protein (Figure 3(B)).

### Purification of VHSVG-FcK protein from transgenic tobacco plant

**Figure 4.**
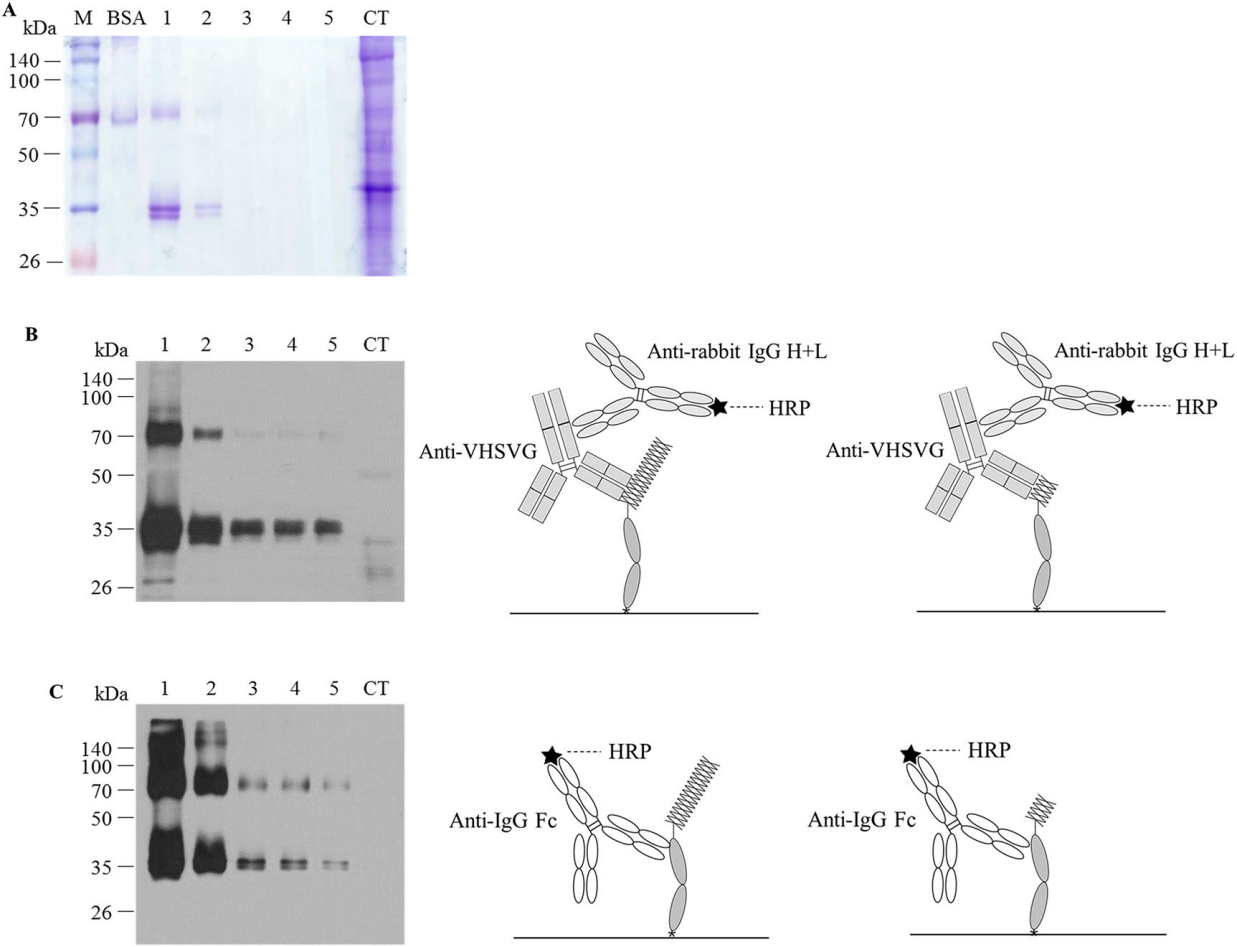
Purification of VHSVG-FcK protein from plant biomass production. (A) SDS-PAGE for detection of VHSVG-FcK protein from purified samples. M, protein marker; BSA, 2 μg; 1–5, eluted fraction sample number; CT, Column through sample. Each sample was loaded with 16 μl, respectively. (B) Immunoblot analysis was performed to confirm the presence of VHSVG-FcK proteins from the eluted samples. VHSVG-FcK (>70 kDa) was detected with rabbit anti-VHSV antibodies. Lane 1–5, fraction number 1–5 of purified samples; CT, Column through sample. The sample was loaded 16 μl, respectively. (C) Immunoblot analysis was conducted with HRP-conjugated goat anti-human IgG Fc antibodies. Lane 1–5, fraction number 1–5 of purified samples; CT, Column through sample. The sample was loaded 8 μl, respectively.

VHSVG-FcK protein was purified from leaves produced from mass production of tobacco plant by using protein A affinity chromatography (Figure 2). SDS-PAGE was performed with eluted fraction samples to observe the purified protein band (Figure 4(A)). The expected protein band of VHSVG-FcK was detected at >70 kDa in the fraction samples #1 and 2. Non-specific total soluble protein bands were detected in column. However, unexpected protein bands were also detected at around 35 kDa (Figure 4(A)). Immunoblot analysis with both anti-VHSVG antibody and anti-IgG Fc antibody was performed to confirm the specific VHSVG-FcK protein band in each eluted fraction (Figure 4(B,C), respectively). The VHSVG-FcK protein band was detected at approximately >70 kDa in the fraction 1–5 samples by both antibodies. The 35 kDa protein band also was detected in the fraction 1–5 samples. >70 and 35 kDa bands were not detected in the column through (CT).

### Immune response of mice against plant-derived subunit vaccine candidate VHSVG-FcK

**Figure 5.**
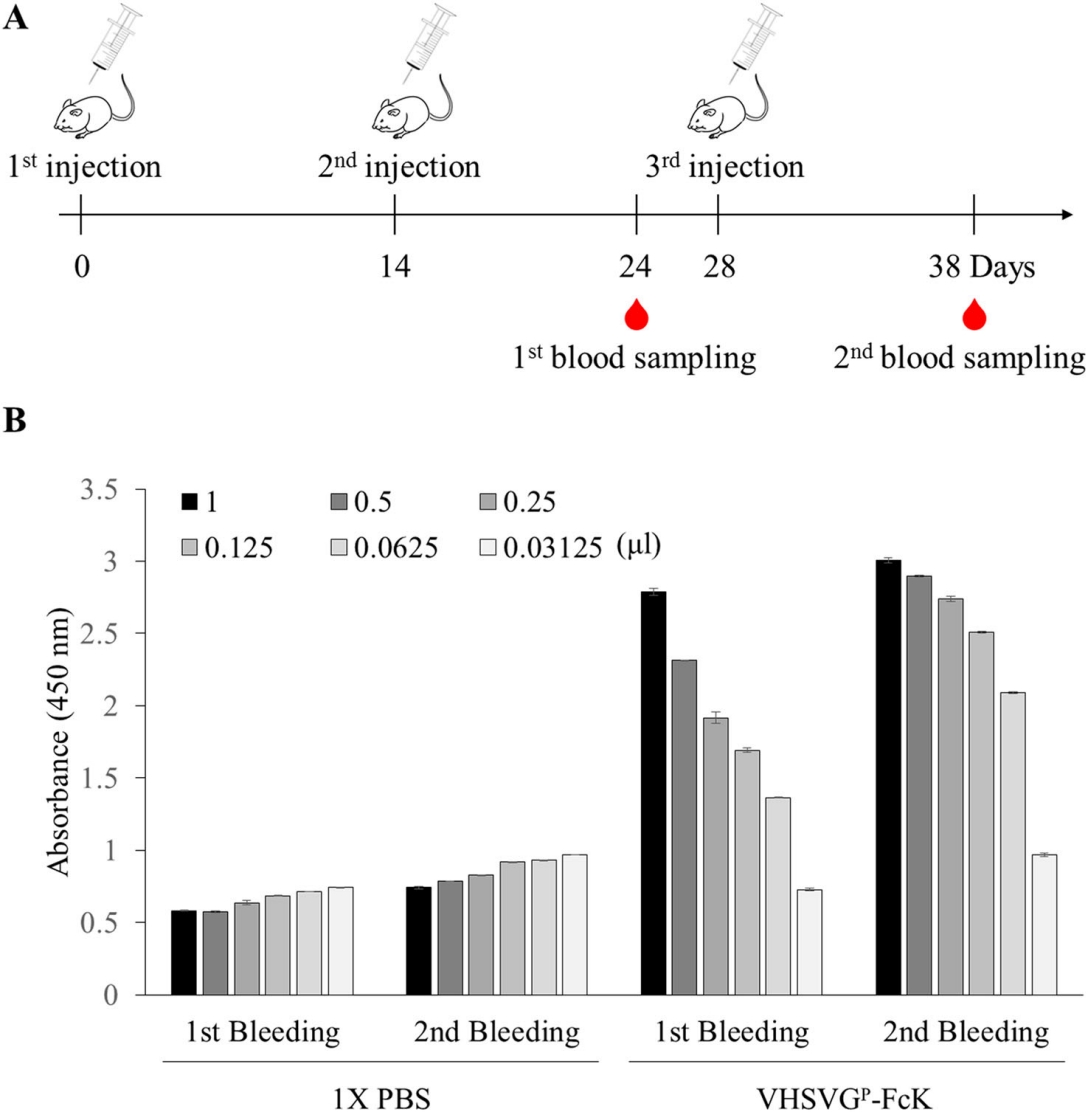
Schematic diagram of the immunization of VHSVG-FcK in mice and ELISA to confirm immune response of VHSVG-FcK in mice. (A) VHSVG^P^-FcK was injected to eight-week-old female BALB/c mice three times via intraperitoneal injection. 1X PBS was administered as a negative control using the same injection method. The 10 μg of VHSVG^P^-FcK and 1X PBS in a total volume of 300 μl were injected with an adjuvant (aluminum hydroxide). The samples were injected at intervals of 2 weeks and blood samples were collected 10 days after the second immunization and 10 days after the third immunization by the retro-orbital sinus bleeding method. (B) ELISA result of 1X PBS and VHSVG-FcK immunization. Ninety-six-well plate wells were coated with VHSVG-FcK (100 ng/well), applied with mouse serum as a capture antibody, and treated with HRP-conjugated anti-mouse IgG Fc.

ELISA was performed to confirm the specific antibodies production against the VHSVG-FcK in mice after the plant-derived VHSVG-FcK injection (Figure 5). The sera collected from mice at 10 days after the 2nd injection and 10 days after the 3rd injection (1st and 2nd bleed samplings, respectively) were applied to a 96-well plate coated with 100 ng of VHSVG^P^-FcK (Figure 5(A,B)). The secondary antibody used was HRP-conjugated anti-mouse IgG Fc to detect the VHSVG^P^-FcK specific antibodies in the mice serum. The absorbance of the mice injected with the VHSVG^P^-FcK group was significantly higher than the mice injected with 1X PBS. The absorbance in the 2nd bleeding sample was slightly higher than that in the 1st. In both 1st and 2nd bleeding samples, the absorbances were sequentially decreased along with the decreasing serum amount added to the plates.

## Discussion

In this study, we successfully established a plant expression system for the production of the recombinant VHSVG-FcK fish vaccine candidate. Although many immunotherapeutic proteins have been produced in plant expression systems that are safe, cost efficient, and easy to scale up, expression of fish vaccines has not been studied extensively in plants. The present study demonstrated that the recombinant subunit vaccine protein VHSVG-FcK as a fish vaccine candidate was expressed in the tobacco plant, and this plant-derived VHSVG-FcK purified from tobacco plant leaves induced an immune response in mice.

The subunit vaccine candidate, VHSVG, was fused to the immunoglobulin Fc fragment and cloned under the control of the enhanced CaMV 35S promoter with AMV. Fusion of Fc to the recombinant subunit vaccine endows the molecules to interact with Fc-receptors (FcRs) on immune cells (Nimmerjahn and Ravetch 2007). Thus, it is expected that the Fc-region bound to Fc-receptors should efficiently prepare antigen delivery vehicles to antigen presenting cells (DiLillo and Ravetch 2015). In addition, the Fc region can be applied in protein-G/A affinity chromatography to purify the Fc fused recombinant protein from plant leaf biomass (Lu et al. 2012). Our previous studies showed that the various antigenic proteins fused to the Fc fragment expressed in plant (Lee and Ko 2017) and insect cell (Moussavou et al. 2018) were efficiently purified by protein A or G affinity chromatography column (Flanagan et al. 2007; Czajkowsky et al. 2012). The function of KDEL-tagging is to retain the tagged protein to ER from cis-Golgi, eventually enhancing protein accumulation levels (Tekoah et al. 2004). Thus, the KDEL (Lys-Asp-Glu-Leu) ER retention motif was fused to enhance protein expression level and retain the recombinant vaccine, inducing oligo-mannose type of glycan structures to relieve immune response in other species (Figure 1). The ER-retained proteins have typically Man_8_GlcNAc_2_ (Man8) N-glycans (Gomord et al. 2010). However, once exposed to cis-Golgi processing enzymes such as α (1,2)-mannosidase, the KDEL-tagged proteins carry Man7 and Man6 N-glycans, which are partially trimmed N-glycans (De Meyer and Depicker 2014).

To confirm the VHSVG-FcK gene insertion, PCR analysis was conducted. Some regenerants did not undergo transgene insertion. These results are consistent with previous studies where some regenerants had nptII genes but not the targeted transgene of interest (Ko et al. 2003). The immunoblot analysis result confirmed VHSVG-FcK protein expression in all tested plants with transgene insertion. The intensity of each band was different, indicating that expression levels by each transgenic plant varied. The variation of transgene expression has been reported in many studies. The variation of transgene expression is due to the position effect and copy number (Matzke and Matzke 1998; Kooter et al. 1999). For further study, T810 with the highest expression was chosen for leaf biomass production. A non-specific protein band (35 kDa) was also detected in all samples. According to the detected protein band size (35 kDa) by both anti-Fc and anti-VHSVG antibodies, it is speculated the cleaved fragment (35 kDa) consists of partial fragments of the VHSVG and Fc region

Both SDS-PAGE and immunoblot analyses were performed to investigate whether the VHSVG-FcK was successfully purified using protein A affinity chromatography. The expected VHSVG-FcK band (80 kDa) was detected in the fraction samples #1 and 2. However, the unexpected protein band (35 kDa) was also detected. The band pattern was similar to that produced by anti-human IgG Fc antibody conjugated to HRP in immunoblot analysis. Since protein A can bind to the Fc region of immunoglobulin, it is speculated that the detected 35 kDa protein bands are only FcK fragments. However, both anti-VHSG and anti-human IgG Fc antibodies detected the 35 kDa proteins, suggesting that the 35 kDa protein is composed of the C-terminus part of the VHSV and FcK domains.

The immunological properties of VHSVG^P^-FcK after the second or third injections in mice were investigated. The VHSVG domain has not been studied as a target for anti-virus recombinant vaccines. Thus, it is crucial to investigate whether the VHSVG domain can induce immune responses. Indeed, in the current study, the VHSVG domain fused to the Fc region induced immune responses. Immunization with viral antigen is a potential strategy for prevention against fish virus infection (Plant and LaPatra 2011). ELISA was performed to detect the specific antibodies produced against the VHSVG^P^-FcK injected in mice. The second bleeding sample, after the third injection, showed the stronger absorbance signal suggesting that the third injection boosted the immune responses. These results were consistent with previous studies where the third injection with anti-cancer antigen as a vaccine candidate induced immune responses compared to the first and second injections in mice (Lee et al. 2016). The VHSVG^P^-FcK injection group showed notable absorbance compared to the 1X PBS injection group as a negative control. The immune responses seemed to produce anti-human Fc IgGs. According to the western blot with serum from the VHSVG-FcK injected mice (Supplemental Figure 1), the human IgG heavy chains (HC) were detected by the serum. However, the density of VHSVG-FcK protein bands detected by the serum was stronger than the HC band of human mAb, indicating that the VHSVG-FcK induces more anti-VHSVG IgG immune responses compared to the Fc fragments in mice. Taken together, these results suggest that the recombinant VHSVG-FcK vaccine candidate can have an immunogenicity.

In conclusion, our results showed that the recombinant VHSVG-FcK subunit vaccine protein can be expressed in transgenic plants, purified using protein A affinity chromatography, and evoke the immune response in mice. Fusion of VHSV domain to the human IgG Fc fragment is an ideal strategy to purify the recombinant vaccine candidate protein expressed in transgenic plants. However, the stability of VHSVG-Fc fusion protein without degradation and cleavage still remains to be determined. Nevertheless, a plant expression system can be applied to develop recombinant fish vaccine.

## Supplementary Material

Supplemental Material
