# Supplementary material for: Immune response of heterologous recombinant antigenic protein of viral hemorrhagic septicemia virus (VHSV) in mice
Source: Anim Cells Syst (Seoul). 2019 Feb 8;23(2):97–105. doi: 10.1080/19768354.2019.1575904 (PMC6440531; doi:10.1080/19768354.2019.1575904)
Supplement: Supplemental Material [file TACS_A_1575904_SM9664.docx]

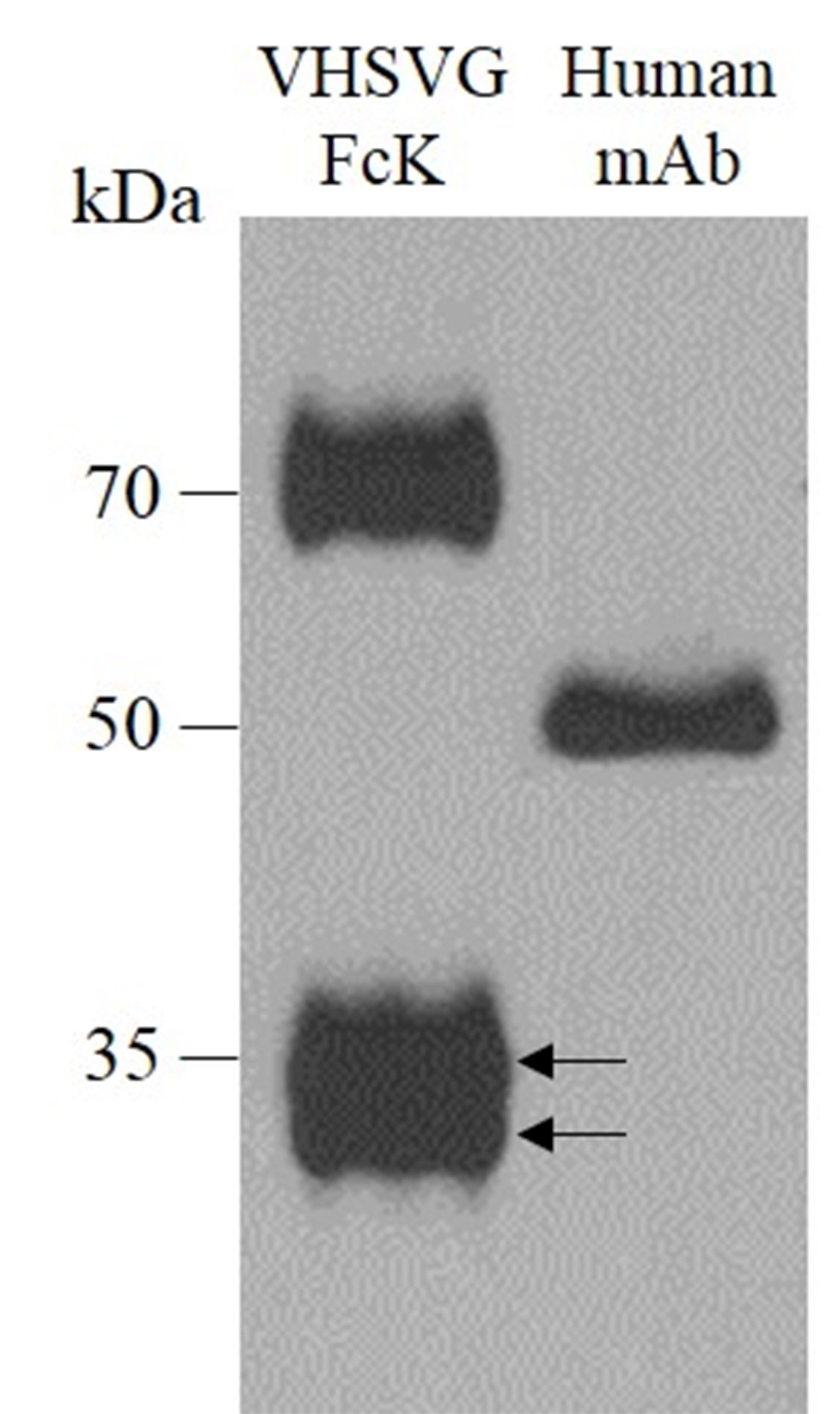


Supplemental Figure 1. Confirmation of anti-human IgG Fc existence in VHSVG-FcK injected mice serum. Immunoblot analysis was performed to confirm whether serum from mice injected with VHSVG-FcK has anti-human Fc IgGs. The 10 μl of mice serum from VHSVG-FcK injection group was treated as a primary antibody, and HRP-conjugated goat anti-mouse IgG Fc antibody was applied as a secondary antibody (1:5,000). Lane 1, purified VHSVG-FcK sample; Lane 2, human mAb. The 100 ng of samples were loaded respectively. We found that the VHSVG-FcK fish vaccine can be expressed in plant and induce immunogenicity in mice. We did western blot analysis to confirm whether serum from mice injected with VHSVG-FcK has both anti-VHSVG and anti-human Fc IgGs. As shown in the above Supplemental Figure 1, the serum detected the Fc of human IgG. The serum detected two bands around 35 kDa which are VHSVG and Fc fragments (arrows). This result indicates the VHSVG-FcK can induce both VHSVG and human Fc fragments. However, the density of VHSVG-FcK proteins detected by the serum were stronger than the heavy chain fragment itself of human mAb. This result indicates that the VHSVG-FcK induces more anti-VHSVG fragments in mice compared to the Fc fragments.
